# Regulation of Cancer Metastasis by TRAIL/Death Receptor Signaling

**DOI:** 10.3390/biom11040499

**Published:** 2021-03-26

**Authors:** You-Take Oh, Shi-Yong Sun

**Affiliations:** Department of Hematology and Medical Oncology, Emory University School of Medicine and Winship Cancer Institute, Atlanta, GA 30322, USA; youtake.oh@emory.edu

**Keywords:** TRAIL, death receptor, apoptosis, invasion, metastasis, cancer

## Abstract

Death ligands such as tumor necrosis factor-related apoptosis-inducing ligand (TRAIL; TNFSF10) and their corresponding death receptors (e.g., DR5) not only initiate apoptosis through activation of the extrinsic apoptotic pathway but also exert non-apoptotic biological functions such as regulation of inflammation and cancer metastasis. The involvement of the TRAIL/death receptor signaling pathway in the regulation of cancer invasion and metastasis is complex as both positive and negative roles have been reported. The underlying molecular mechanisms are even more complicated. This review will focus on discussing current knowledge in our understanding of the involvement of TRAIL/death receptor-mediated signaling in the regulation of cancer cell invasion and metastasis.

## 1. Introduction

Metastasis is a key cause of cancer death. To metastasize to distant organ sites, cancer cells must detach from the primary tumor sites (local invasion; intravasation), translocate into the circulatory system, survive in the circulation and arrest at a distant organ site (extravasation), and finally adapt to the new microenvironment of distant tissues (micrometastasis formation; metastatic colonization) [1]. Therefore, cell death is a key mechanism for preventing the dissemination of metastatic cells. Cell death can occur after the detachment of primary tumor cells from the extracellular matrix, in the circulation through tumor immune surveillance or destruction by mechanical stresses, and after extravasation during the phase of micrometastasis formation in a secondary organ [2]. It has been estimated that only about <0.01% of cancer cells that enter the circulation are able to eventually survive and develop lung metastasis [3,4]. This makes metastasis a highly inefficient process: very few of the cancer cells that migrate from the primary tumor can successfully colonize in distant sites [1].

Apoptosis represents a major form of cell death. Thus, the acquisition of apoptosis resistance is a key hallmark of cancer and occurs early during tumorigenesis [5]. Accordingly, it seems logical that the selection of cancer cells with greater deregulation of apoptosis will be necessary for strong metastasis. It is well known that the extrinsic death receptor-mediated apoptotic pathway plays a critical role in host immune surveillance against cancer [6,7,8]. However, an increasing body of research suggests that this death signaling pathway also exerts non-apoptotic functions in enhancing cancer growth or metastasis. This review will primarily focus on discussing current knowledge in our understanding of the involvement of the extrinsic apoptotic pathway, particularly tumor necrosis factor-related apoptosis-inducing ligand (TRAIL)/death receptor-mediated signaling, in the regulation of cancer cell metastasis.

## 2. TRAIL/Death Receptor-Mediated Apoptotic Signaling Pathway

**Figure 1:**
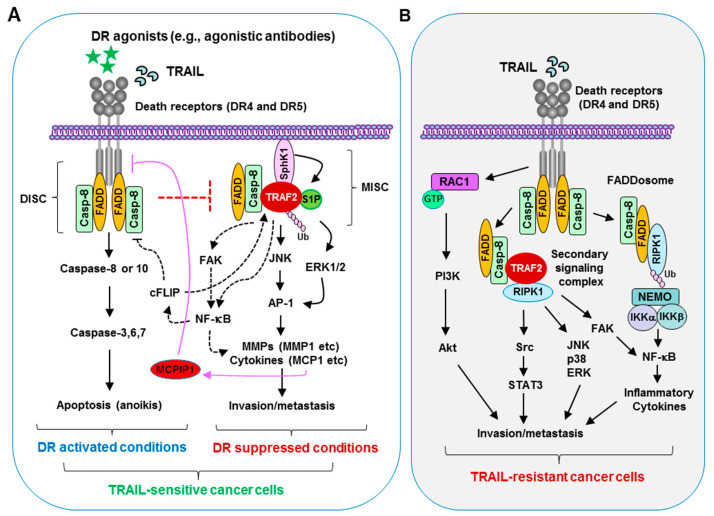
Tumor necrosis factor-related apoptosis-inducing ligand (TRAIL)/death receptor signaling in the regulation of apoptosis and metastasis of cancer cells. (**A**) Under normal apoptotic conditions (e.g., in cancer cells sensitive to TRAIL with activated death receptor pathway), TRAIL ligation with its death receptors (DR4 and DR5) or an agonistic antibody binding to a corresponding death receptor (DR4 or DR5) on the surface of cancer cells induces the formation of the death-inducing signaling complex (DISC) involving Fas-associated death domain (FADD) recruitment of pro-caspase-8 via its death effector domain, resulting in caspase-8 or -10 activation followed by cleavage and activation of caspase-3, -6, and -7, and eventual execution of apoptosis or anoikis. This mechanism restricts the formation of the metastasis and invasion signaling complex (MISC) and subsequently suppresses cell invasion and metastasis. When TRAIL/death receptors are inhibited or their functions are compromised, available FADD and caspase-8 may recruit and stabilize TNF-receptor-associated factor (TRAF)2 with the help of S1P, resulting in enhanced TRAF2 polyubiquitination and activation, likely through a self-ubiquitination mechanism. This will further lead to activation of ERK/JNK/AP-1 signaling and NF-κB activation, which activates MMPs (e.g., MMP1) and enhances the release of inflammatory cytokines (e.g., monocyte chemoattractant protein 1 (MCP1)) that promote invasion and metastasis of cancer cells. MCP1 may induce monocyte chemotactic protein-induced protein-1 (MCPIP1) expression, leading to a reduction in DR5 levels, including cell surface DR5; this favors MISC formation and metastasis. (**B**) In TRAIL-insensitive cells, TRAIL treatment will induce the formation of a second signaling complex or FADDosome, resulting in the activation of multiple protein kinases such as NF-κB, JNK, p38, ERK, and Src that are involved in the positive regulation of invasion and metastasis of cancer cells. In *KRAS*-mutated cancer cells, TRAIL/DR5 activation can activate the Rac1/PI3K/Akt axis, promoting cell invasion and metastasis. RIPK1, receptor-interacting serine/threonine-protein kinase 1. DR, death receptor.

There are two TRAIL death receptors in humans: death receptor 5 (DR5; also called TRAIL-R2, TNFRSF10B or Killer/DR5) and death receptor 4 (DR4; also called TRAIL-R1 or TNFRSF10A). Both DR5 and DR4 share redundant functions in triggering apoptosis but have some distinct biological functions, such as regulation of cancer cell metastasis, as discussed below. In contrast, there is only one TRAIL death receptor named murine TRAIL death receptor (mDR) in mice [9,10] or mouse homolog of KILLER/DR5 (MK) [11]. TRAIL ligation to its functional cell surface death receptors or induction of death receptor trimerization or aggregation (e.g., via overexpression or agonistic antibodies) leads to recruitment of the adaptor protein, Fas-associated death domain (FADD), to the cytoplasmic region of the receptor followed by recruitment of pro-caspase-8 or pro-caspase-10. The formation of this death-inducing signaling complex (DISC) triggers cleavage and activation of caspase-8 or caspase-10, which in turn activates downstream caspase-3, -6, and -7, and eventual apoptosis [12,13]. Therefore, TRAIL/death receptor/FADD/caspase-8 signaling normally triggers apoptotic cell death (Figure 1A).

The cellular FLICE-like inhibitory protein (c-FLIP) is a truncated and dominant-negative homolog of caspase-8 and functions to inhibit death receptor-mediated apoptosis by competing with caspase-8 for binding to FADD [14,15]. FLIP, which is expressed as short (FLIP_S_) and long (FLIP_L_) splice forms, has two death effector domains and can allow the recruitment of caspase-8. The C-terminal region of FLIP_L_ contains a caspase homolog that lacks caspase activity. Formation of the pro-caspase-8/FLIP_L_ heterodimer complex can limit caspase-8 activation. In FLIP_S_, there is no such homolog of caspase; instead, there is a short region of 20 amino acids responsible for its ubiquitylation and proteasomal degradation. Both forms of FLIP can compete for caspase-8 homodimer complex assembly, and so, when FLIP is heterodimerized with caspase-8, it inhibits caspase-8-dependent cell death [16,17].

Epithelial cells normally undergo detachment-induced apoptosis, termed anoikis, once losing anchorage to the extracellular matrix or when adhesion to the correct substrate is disrupted, primarily by triggering the death receptor-mediated extrinsic apoptotic pathway. In contrast, malignant epithelial cells with metastatic potential resist anoikis and can survive in an anchorage-independent fashion [18,19].

## 3. TRAIL/Death Receptors in Negative Regulation of Cancer Metastasis

### 3.1. Critical Role of TRAIL Produced by Immune Cells in Negative Regulation of Tumor Development and Metastasis

TRAIL is expressed in various tissues, particularly immune cells [20], and plays a critical role in NK or T cell-mediated immune surveillance against tumor development and metastasis [21]. Earlier studies suggested that TRAIL can suppress experimental liver metastasis or contribute to interferon (INFγ)-mediated anti-metastatic effects [22,23]. When neutralizing monoclonal antibody against TRAIL was administered, significantly increased experimental liver metastases of several TRAIL-sensitive tumor cell lines were observed. Such an anti-metastatic effect of TRAIL was not observed in NK cell-depleted mice or INFγ-deficient mice, the latter of which lacked TRAIL in liver NK cells [22]. Consistently, administration of therapeutic doses of interleukin (IL)-12, a powerful inducer of IFNγ production by NK cells and NKT cells, upregulated TRAIL expression in the liver, spleen, and lung NK cells, and suppressed metastases in both the liver and lungs in a TRAIL-dependent fashion [23]. To further demonstrate the important role of TRAIL in suppressing tumor initiation and metastasis, TRAIL gene-targeted mice were generated, which lacked TRAIL in liver and spleen mononuclear cells. These TRAIL gene-targeted mice were more susceptible to experimental and spontaneous tumor metastasis and also more sensitive to the chemical carcinogen methylcholanthrene, further supporting TRAIL as an important natural effector molecule used in the host defense against transformed cells and cancer cell metastasis [24]. These early studies strongly suggest that TRAIL expressed in immune cells, such as NK cells, contributes to the natural suppression of tumor development and metastasis, likely via TRAIL-mediated apoptosis.

### 3.2. TRAIL-Targeted Therapeutic Approaches Against Metastatic Cancers

Based on this rationale, therapeutic strategies against metastatic cancer cells have been explored. In a breast cancer xenograft model, injection of TRAIL-expressing CD34⁺ cells, CD34-TRAIL(+) cells, did not lead to tumor growth inhibition; however, lungs in the tumor-bearing mice were completely free of metastases at 12 days after the last injection of CD34-TRAIL(+) cells, whereas metastases were present in all control mouse lungs [25]. In a renal cell carcinoma orthotopic tumor model with aggressive primary tumors and lung metastases detectable by day 7, intra-renal administration of Ad5mTRAIL+CpG, an adenovirus-encoded TRAIL/CpG immunotherapy regimen, on day 7 led to an influx of effector phenotype CD4 and CD8 T cells into the kidney by day 12 and regression of established primary renal tumors. Moreover, systemic immune responses characterized by splenomegaly, elevated serum IgG levels, increased CD4 and CD8 T cell infiltration into the lungs, and elimination of metastatic lung tumors were also detected [26]. A recent study reported that TRAIL-modified adipose-derived stem cells (ADSCs) could migrate toward hepatocellular carcinoma (HCC) cells to inhibit tumor growth and the metastasis of implanted HCC tumors [27]. It was also shown that TRAIL-coated leukocytes effectively killed cancer cells in the circulation, mimicking the activity of NK cells to neutralize circulating tumor cells that enter blood with the potential to form new metastases [28]. This approach was further shown to prevent the spontaneous formation and growth of metastatic tumors in an orthotopic xenograft model of prostate cancer by greatly reducing the number of circulating tumor cells [29]. In agreement, minimal dosing of leukocyte-targeting TRAIL via administration of E-selectin-TRAIL liposomes decreased triple-negative breast cancer metastasis following tumor resection in a preclinical mouse model [30].

It has been shown that tumor cell lines derived from metastatic lymph nodes displayed a greater TRAIL resistance than cell lines derived from the respective primary oral tumors [31]. Consistently, TRAIL gene therapy alone or in combination with chemotherapy decreased the number of lung metastases from both chemosensitive and chemoresistant breast cancer cell lines. The combination of TRAIL gene therapy and chemotherapy resulted in a further reduction of lung metastatic nodules with minimal toxicity [32]. Co-administration of soluble TRAIL with anticancer drugs inhibited liver metastasis of TRAIL-resistant colorectal cancer cells [33]. Another study reported that recombinant TRAIL treatment marginally prevented metastatic spread because liver metastases were detected in 71% of mice, whereas 86% of vehicle-treated mice developed liver metastases; however, treatment with APG350, a TRAIL-receptor agonist and potent inducer of apoptosis, inhibited liver metastases by 29% [34]. These studies further document the anti-metastasis function of activating the TRAIL/death receptor pathway. In a model for metastatic colon cancer, tail vein infusion of a tumor-targeted and conditionally replicating oncolytic adenovirus vector expressing TRAIL (Ad5/35.IR-E1A/TRAIL) resulted in the elimination of pre-established liver metastases [35]. Administration of a different oncolytic adenovirus encoding TRAIL (P55-HTERT-HRE-TRAIL) also significantly reduced orthotopic breast tumor growth and extended survival in a metastatic model [36]. In another study using telomerase-specific oncolytic adenovirus expressing TRAIL, it was shown that this approach significantly inhibited peritoneal metastasis of gastric cancer and prolonged the survival of mice without treatment-related toxicity, indicating effective suppression of peritoneal dissemination of gastric cancer cells [37]. Similarly, recombinant adeno-associated virus-mediated TRAIL gene therapy suppressed liver metastatic tumors [38]. These studies suggest the potential of using these means for gene therapy of metastatic cancer. More recently, the TRAIL-inducing small molecule, ONC201, which is being tested in clinical trials, was shown to effectively inhibit cancer metastasis and promote intratumoral NK cell recruitment, including stimulation of an increase in activated TRAIL-secreting NK cells in the peripheral blood of patients [39].

### 3.3. Critical Role of TRAIL Death Receptor Expression in Negative Regulation of Cancer Metastasis

Early studies using a mouse knockout model suggest that mDR plays a suppressive role in cancer metastasis without affecting primary tumor growth since mDR knockout significantly increased lymph node metastasis of carcinogen-induced skin carcinomas [40] and metastasis of lymphoma cells to the liver and lungs during *MYC*-driven lymphomagenesis [41]. In agreement, our study using human cancer cells showed that inhibition of DR5 by knockdown or knockout increased invasion of several human cancer cell lines and significantly increased lung metastasis of cancer cells in a nude mouse carrying subcutaneous lung cancer xenografts [42]. Moreover, DR5 agonistic antibody lexatumumab was shown to robustly suppress lymph node or lung metastasis in an orthotopic model of triple-negative breast cancer [43]. Similar results were also generated with another DR5 agonist named MEDI3039 [44]. These preclinical studies strongly suggest that DR5 negatively regulates invasion and metastasis of both murine and cancer cells beyond its apoptosis-inducing function.

**Figure 2:**
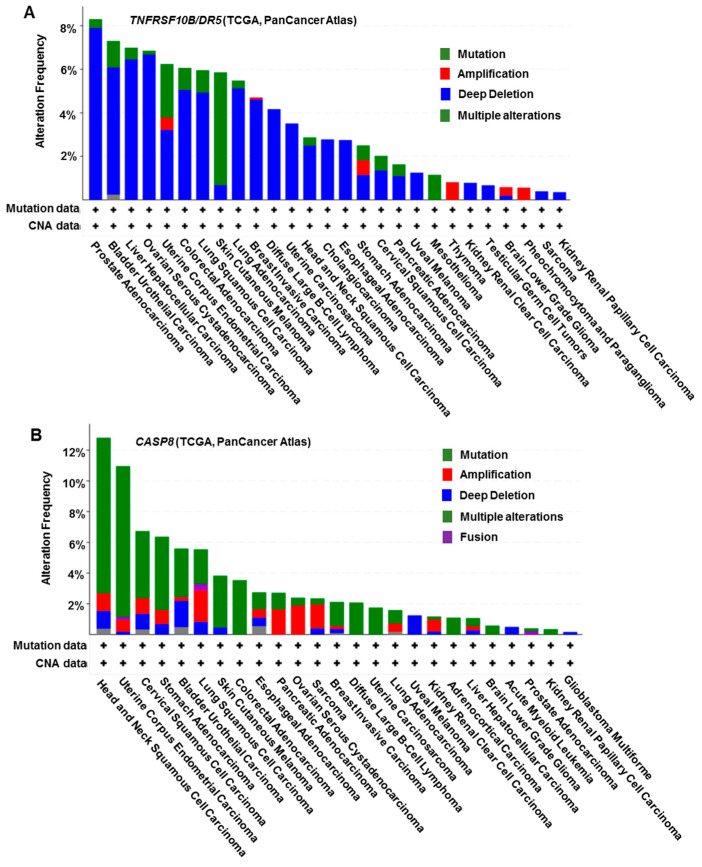
*DR5 (TNFRSF10B)* and *CASP8* gene mutations in different cancer types with TCGA data analysis. These data were generated from the cBioPortal website at https://www.cbioportal.org/ (accessed on 16 March 2021).

Reduced DR5 expression in human melanoma tumor samples was reported to be associated with metastatic lesions [45]. Our own study found that DR5 expression in primary head and neck cancer (HNC) specimens with metastasis and their matching lymph node metastasis was significantly reduced in relation to primary tumors without evidence of metastasis [46]. Intriguingly, inactivating mutations primarily in the death domain of DR5 were detected exclusively in approximately 12% of breast cancer with lymph node metastasis, but not in breast cancer without metastasis [47]. Mutations in DR5 might alter DR5-mediated apoptosis and metastasis, albeit with varied low frequencies in many types of cancer in general (Figure 2A). These findings from human cancer tissues also support the role of DR5 in the suppression of cancer metastasis.

In the early stage of HNCs with no lymph node metastasis, we found that higher expression of caspase-8 alone or in combination with higher DR5 expression significantly correlated with better disease-free survival and overall survival [46], suggesting an inhibitory role of DR5/caspase-8 signaling in the regulation of cancer development and progression. However, in later stage HNCs with lymph node metastasis, high caspase-8 and DR5 together was significantly associated with poor disease-free and overall survival [46]. This is also true for HNCs with lymph node metastasis that possess high FADD expression alone or in combination with high DR5 or caspase-8 expression [48]. These findings suggest that DR5/FADD/caspase-8 signaling may have a role in promoting cancer metastasis in the late stage of cancer (e.g., metastasis). It is possible that the DR5/FADD/caspase-8 signaling in primary HNCs predominantly contributes to activation of apoptosis (e.g., anoikis), resulting in preventing metastasis, but in metastatic HNCs that may have already escaped from apoptosis (i.e., are resistant to anoikis), this signaling may be converted to pro-metastatic signaling, causing promotion of invasion and metastasis.

## 4. TRAIL/Death Receptors in Positive Regulation of Cancer Metastasis

Despite the suppressive function of TRAIL/death receptor in the regulation of cancer cell invasion and metastasis as discussed above, TRAIL has also been reported to strongly induce the expression of pro-inflammatory cytokines interleukin-8 and monocyte chemoattractant protein 1 (MCP1), to enhance the invasion of apoptosis-resistant pancreatic ductal adenocarcinoma (PDAC) cells in vitro by upregulation of the urokinase-type plasminogen activator expression, and to strongly increase the distant metastatic spread of pancreatic tumors in vivo [49]. Similarly, TRAIL was also shown to induce cell migration and invasion in apoptosis-resistant cholangiocarcinoma cells [50]. TRAIL, like CD95 ligand, could stimulate invasion of colorectal tumor cells and liver metastases in a K-Ras-dependent fashion. Loss of mutant K-Ras switched TRAIL receptors back into apoptosis mode and abrogated metastatic potential [51]. It was suggested that levels of soluble TRAIL in human plasma were approximately 28 pg/mL [52], a concentration at which TRAIL is insufficient for effective induction of apoptosis in lung cancer, colorectal cancer, and pancreatic cancer [53]. Beyond acquiring resistance to TRAIL, some circulating TRAIL-responsive cancer cells under such conditions may survive TRAIL-mediated killing and eventually metastasize to distal sites.

Bioinformatic analyses of patient data from 839 adenocarcinoma (AC) and 356 squamous cell carcinoma (SCC) of lung cancer cases by cBioPortal (genomic analyses) showed that TRAIL expression leads to differential outcomes of disease-free survival in AC and SCC. Oncomine datamining (transcript analyses) revealed that TRAIL is upregulated in 167 SCC as compared to 350 AC patients from six data sets. Genomic analyses using cBioPortal revealed high rates of *KRAS* mutation in AC accompanied by a higher incidence of metastasis and increased amplification of the TRAIL gene in SCC. Bioinformatic analyses of an additional lung cancer patient database also showed that the risk of disease progression was significantly increased with high TRAIL expression in AC (461 samples) [54]. Patients with mesenchymal subtype colorectal cancer have a poor prognosis, in particular, patients with stroma-rich tumors and aberrant SMAD4 expression. A recent study suggests that TRAIL produced from SMAD4-deficient colorectal cancer cells induces BMP2 in fibroblasts, which enhances invasiveness and metastasis of colorectal cancer [55].

There was a study demonstrating that mDR and human DR5 promote K-Ras-driven cancer progression, invasion, and metastasis [56]; these results are contradictory to their previous findings using an H-Ras-driven skin carcinogenesis model [40] and to our findings with several human cancer cell lines such as A549, H460, and HCT116, which all have mutant K-Ras [42]. In this study, cancer cell-restricted genetic ablation of mDR in autochthonous *KRAS*-driven models of non-small cell lung cancer (NSCLC) and PDAC was shown to reduce tumor growth, blunt metastasis, and prolong survival by inhibiting cancer cell-autonomous migration, proliferation, and invasion. Moreover, this study detected DR5 expression in human PDAC and colorectal cancer tissues and found that high DR5 expression correlated with invasion of human PDAC into lymph vessels and with shortened metastasis-free survival of *KRAS*-mutated colorectal cancer patients [56]. Mechanistically, the study suggested that constitutive signaling from DR5 promotes activation of a Rac1/PI3K/Akt signaling axis that increases migration and invasion in a cancer cell-autonomous manner [56].

In a different study using an osteotropic variant of MDA-MB-231 breast cancer cells, knockdown of DR5 increased the levels of E-cadherin and decreased migration with strongly impaired ability to form bone metastases in vivo after intracardiac injection. Evaluating possible underlying mechanisms revealed a strong downregulation of CXCR4, the receptor for the chemokine SDF-1 important for homing of cancer cells to the bone. In accordance, cell migration toward SDF-1 was significantly impaired by DR5 knockdown. Conversely, overexpression of DR5 upregulated CXCR4 levels and enhanced SDF-1-directed migration [57]. This study supports the metastasis-promoting function of DR5, albeit using a single cell line model. In another study, DR5 knockdown in pancreatic cancer cells decreased local relapses and the number of macroscopic liver metastases after primary tumor resection in an orthotopic PDAC model. However, the number of micrometastases was increased. The outgrowth of liver metastases was suggested to be associated with increased liver inflammation induced by resection of primary PDAC [58]. This study also suggests a potential role of DR5 in the positive regulation of cancer cell metastasis under specific conditions.

## 5. FADD, Caspase-8, and c-FLIP in Regulation of Cancer Metastasis

FADD and caspase-8 are key proteins in transmitting death receptor-mediated apoptotic signaling, whereas c-FLIP is the key inhibitor of this death signaling. However, there are critical roles of these proteins in the regulation of cancer cell invasion and metastasis; some may be largely independent of death receptors [59,60,61].

### 5.1. FADD in Regulation of Cancer Metastasis

High FADD expression was associated with an increased rate of lymph node metastasis of HNCs and with a shorter distant metastasis-free interval when lymph node metastases were present [62]. Consistently, we also detected a significant increase in FADD expression in primary HNC tumors. Lower FADD expression was significantly associated with better disease-free survival and overall survival in HNC patients with lymph node metastasis, although FADD expression did not significantly affect the survival of HNC patients without lymph node metastasis [48]. In another study with samples from a cohort of Taiwanese HNC patients with oral squamous cell carcinomas, both FADD gene copy number amplification and high protein expression were significantly associated with lymph node metastasis. Patients with both FADD copy number amplification and high protein expression had the shortest disease-free survival and overall survival [63]. Focal adhesion kinase (FAK) is an integrin-associated protein tyrosine kinase that is frequently overexpressed in advanced human cancers and may support tumor growth and metastasis [64]. Both FADD and FAK were reported to be overexpressed in human melanoma tissue. In murine melanoma cell lines, miR-7a, a tumor suppressor that prohibits cell migration and invasion, downregulated FAK expression. When FADD was overexpressed, miR-7a was inhibited and subsequently enhanced FAK expression. FADD interference could reduce the rate of cell migration, which could be rescued by inhibiting miR-7a expression [65]. These studies suggest the connection between FADD overexpression and potential cancer metastasis.

### 5.2. Caspase-8 in Regulation of Cancer Metastasis

Although caspase-8 was reported to be associated with FAK and other proteins, resulting in enhancement of cleavage of focal adhesion substrates and subsequent promotion of cancer cell migration and metastasis under apoptosis-compromised conditions [66], caspase-8 may primarily function as a negative regulator of cancer metastasis. Dysregulation of caspase-8 was suggested to contribute to apoptotic escape and to associate with malignancy and metastasis of cancers. The loss of caspase-8 expression occurs very frequently in neuroendocrine cancers such as neuroblastoma, medulloblastoma, and glioblastoma [67]. The suppression of caspase-8 expression potentiates neuroblastoma metastases, and reconstitution of caspase-8 in deficient neuroblastoma cells suppressed their metastases [68]. Consistent with these results, the knockdown of caspase-8 expression in triple-negative MDA-MB-231 breast cancer cells resulted in a significant increase in migration and invasion [69]. Furthermore, the highest number of genetic alterations of caspase-8 has been registered in head and neck, uterine, cervical, and gastric cancers involving somatic, frameshift, and missense mutations [70,71,72] (Figure 2B). Mutations in pro-caspase-8 identified from HNCs inhibit caspase activation and apoptotic cell death following stimulation of TRAIL. In the case of the L105H, S375*, and S386* mutants, it appears possible that the proteins are constitutively associated with FADD in the absence of death receptor stimulation. Notably, these pro-caspase-8 mutants promoted more rapid cellular migration and invasion than the wild-type protein [73]. Low expression of caspase-8 or mutations that block its pro-apoptotic activity may be associated with apoptosis resistance and thereby contribute to cancer metastasis.

### 5.3. c-FLIP in Regulation of Cancer Metastasis

In human cancer samples, a high abundance of c-FLIP was found to be significantly associated with lymph node metastasis in both gastric adenocarcinoma [74] and endometrial carcinoma [75]. Consistently, c-FLIP expression was also significantly higher in the lung metastases than in the primary tumors of osteosarcoma [76]. In a preclinical study, inhibition of FLIP expression with both chemical inhibitors and siRNA increased anoikis and reduced distal tumor formation in a mouse model for circulating prostate cancer metastasis [77], indicating that FLIP inhibition decreases the survival of circulating tumor cells and thereby decreases metastatic tumor formation in distant organs. Collectively, these findings suggest that c-FLIP may play a role in the positive regulation of cancer cell invasion and metastasis. In addition to its anti-apoptotic functions, c-FLIP has been shown to exert other physiological functions related to cell proliferation and tumorigenesis, including metastasis [78]. One possible underlying mechanism is that FLIP interacts with TNF-receptor-associated factors (TRAF) 1 and 2, as well as with the RIP kinases and Raf-1, resulting in activation of the NF-κB and ERK signaling pathways [79,80]. Activation of these signaling pathways may be associated with the role of c-FLIP in enhancing cancer cell invasion and metastasis.

## 6. Possible Molecular Mechanisms Accounting for Distinct Functions of TRAIL/Death Receptors in Regulation of Cancer Invasion and Metastasis

It is apparent that TRAIL/death receptor signaling is involved in both negative and positive regulation of cancer invasion and metastasis. Under apoptosis-compromised conditions, TRAIL/death receptor has a high likelihood to exert non-apoptotic functions, including invasion/metastasis-promoting activity.

### 6.1. Possible Mechanisms Underlying Positive Regulation of Cancer Cell Metastasis by TRAIL/Death Receptor Signaling

It is now known that non-canonical TRAIL/death receptor signaling exists and results in the subsequent activation of various kinases including RIPK1, IκB/NF-κB, mitogen-activated protein kinases (MAPK) p38, JNK, ERK1/2, MAP3K/TAK1, PKC, PI3K/Akt, and Src in cancer cells, particularly under apoptosis-resistant conditions (see review; [81,82]). Whether activation of these kinases contributes to cancer cell invasion and metastasis induced by TRAIL/death receptor is largely undefined. One study identified that the RIPK1/Src/STAT3 axis mediates TRAIL-dependent migration and invasion of TRAIL-resistant NSCLC cells [83]. In addition, NF-κB activation may also contribute to TRAIL-induced migration and invasion of apoptosis-resistant cholangiocarcinoma cells [50]. The small GTPase Rac1 is an important mediator of migration and invasion. In the study in which DR5 promoted K-Ras cancer cell invasion and metastasis, it was suggested that constitutive signaling from DR5 promotes activation of a Rac1/PI3K/Akt signaling axis that increases migration and invasion [56] (Figure 1B).

In addition to the primary DISC formation, TRAIL also induces the formation of a secondary signaling complex that retains the DISC components FADD and caspase-8 but recruits several other factors involved in kinase activation such as RIPK1, TRAF2, and NEMO/IKKγ. This secondary complex formation requires FADD, as well as caspase-8 activity, and activates NF-κB, MAPKs, JNK, and p38 pathways [84]. Therefore, under certain conditions such as apoptosis resistance or suppression, activation of these signaling pathways following the formation of the secondary signaling complex can stimulate the survival/proliferation, migration/invasion/metastasis, and inflammation in a context-dependent manner (Figure 1B).

Although inflammation alone is insufficient for causing cancer or metastasis, the inflammatory response can greatly increase the risk of metastasis through the generation of pro-inflammatory biomolecules (e.g., TNFα, IL6, IL8, and MCP1), sphingosine-1-phosphate (S1P), matrix metalloproteinases (MMPs), and angiogenic factors (e.g., VEGF) at the site of inflammation [85,86,87]. While exerting apoptosis-inducing activity, TRAIL can also induce NF-κB-dependent expression of multiple pro-inflammatory cytokines and chemokines, particularly under apoptosis-compromised conditions [88]. Endogenous TRAIL/death receptor-mediated caspase-8 and FADD-dependent inflammatory cytokine (e.g., CCL2/MCP-1) secretion in some TRAIL-resistant cancer cells promotes the accumulation of tumor-supportive immune cells in the cancer microenvironment, thereby encouraging cancer growth. In this process, induction of the assembly of a similar secondary caspase-8-FADD-RIPK1 inflammatory complex called the FADDosome that leads to the activation of NF-κB-dependent signaling is a critical event [88,89] (Figure 1B). Here, caspase-8 functions as an essential scaffold for the assembly of the complex independent of its caspase activity [88]. Whether this mechanism contributes to enhanced cancer cell invasion and metastasis needs further investigation.

TRAF2 is present in the DISC complex and negatively regulates TRAIL-induced caspase-8 activity [90]. Moreover, it is also a key component in the secondary signaling complex induced by TRAIL that activates different kinases [84]. High TRAF2 expression is associated with tumor metastasis and poor survival in gastric cancer [91]. Overexpression of TRAF2 promotes invasion of pancreatic tumor cells and resistance to CD95-mediated apoptosis [92]. S1P, which promotes the formation of lung premetastatic niches and lung metastasis of breast cancer [93], is a lipid cofactor for TRAF2 biological activity [94]. It has been recently demonstrated that TRAF2 directly interacts with FAK and colocalizes to intracellular cell membranes, promoting NF-κB and cell survival via resistance to anoikis [95]. Hence, it is likely that TRAF2 may play a critical role in the regulation of the survival and metastasis of TRAIL-resistant cancer cells (Figure 1).

### 6.2. Possible Mechanisms Accounting for Negative Regulation of Cancer Metastasis by TRAIL/Death Receptor Signaling

In addition to the positive regulation of cancer cell invasion and metastasis, TRAIL/death receptor signaling is largely involved in the negative regulation of cancer cell migration, invasion, and metastasis, as discussed above. Beyond its apoptosis or anoikis-inducing activity that helps to eliminate circulating metastatic cancer cells to prevent dissemination of cancer cells, activation of the pathway may also exert non-apoptotic functions in the suppression of cancer metastasis. Based on our findings [42,96], we suggest that the activation of DR5 normally favors the formation of the DISC, resulting in induction of apoptosis or anoikis as well as other potential biological consequences; this will not only lead to direct killing of detached cancer cells (e.g., via anoikis or TRAIL/DR5-mediated immune surveillance) but also restrict the formation of another complex named the metastasis and invasion signaling complex (MISC), eventually resulting in the suppression of cancer cell invasion and metastasis. When DR5 is inhibited (e.g., by mutation, deficiency, or reduced expression), cancer cells will become resistant to anoikis/apoptosis or immune surveillance. Available FADD and caspase-8 may recruit and stabilize TRAF2; this process will be enhanced by intracellular S1P (e.g., generated by SphK1). TRAF2 will then be polyubiquitinated and activated, likely through a self-ubiquitination mechanism, leading to the activation of ERK1/2 and particularly JNK signaling and subsequent AP-1-dependent expression and activation of MMPs (e.g., MMP1) and finally, promotion of invasion and metastasis of cancer cells [42,96] (Figure 1A).

Monocyte chemotactic protein-induced protein-1 (MCPIP1; also called Regnase-1) encoded by the ZC3H12A gene critically regulates inflammatory responses and immune homeostasis primarily [97]. As its name implies, it is an MCP1-induced protein. We recently found that MCPIP1 and MCP1 negatively regulate DR5 protein levels, including cell surface DR5 and cancer cell responses to TRAIL, primarily through modulating DUB-mediated protein autophagic/lysosomal degradation [98]. Given that TRAIL induces MCP1 expression in TRAIL-resistant cancer cells [88], it is plausible to speculate that increased MCP1 expression and release is likely to induce MCPIP1 expression; this, in turn, downregulates the levels of DR5 including cell surface DR5, promoting the formation of MISC and subsequent invasion and metastasis of cancer cells (Figure 1A). Thus, further investigation in this direction is warranted.

**Figure 3:**
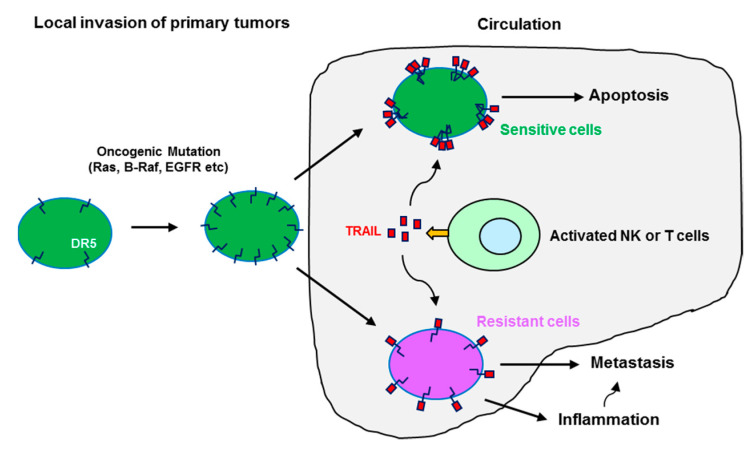
A schematic illustration of the potential impact of oncogenic Ras and Raf mutation-induced DR5 expression on apoptosis and metastasis of cancer cells. While the majority of Ras or Raf mutant cancer cells with elevated DR5 in the circulation will be eliminated by activated immune cells such as NK and T cells that produce TRAIL, triggering TRAIL/DR5-mediated apoptosis through ligation with cell surface death receptors (e.g., DR5) of cancer cells, a subset of TRAIL/DR5-resistant cells with elevated DR5 may survive from escaping the immune surveillance in circulation to eventually form metastases; the elevated DR5 here may favor the formation of metastasis-related signaling complexes such as the FADDosome and enhancement of inflammation and cancer cell invasion and metastasis.

Oncogenic mutations of Ras and B-Raf frequently occur in many cancer types and are critical for cell transformation and tumorigenesis. Interestingly, DR5 has been shown to positively regulate cell invasion and metastasis in *KRAS*-mutated cancer cells [56] while suppressing invasion and metastasis in *HRAS* mutant animal models [40], albeit with an undefined underlying mechanism. We have shown that both Ras and B-Raf induce DR5 expression by enforced expression of oncogenic Ras (e.g., H-Ras12V or K-Ras12V) or B-Raf (i.e., V600E) in cells and by analyzing gene expression array data generated from cancer cell lines and from human cancer tissues. This finding is further validated through knockdown of endogenous K-Ras or B-Raf (V600E), which reduced the expression of DR5. This DR5 induction occurs primarily through co-activation of ERK/RSK and JNK signaling pathways and subsequent cooperative effects among the transcriptional factors CHOP, Elk1, and c-Jun in enhancing DR5 gene transcription. We also showed that the majority of cancer cell lines highly sensitive to the DR5 agonistic antibody AMG655 have either Ras or B-Raf mutations [99]. It is possible that *RAS*- or *RAF*-mutated cells with elevated DR5 expression barely survive in circulation and will be eliminated (e.g., via immune surveillance) if they are sensitive to TRAIL/DR5-mediated apoptosis. A subset of *RAS*- or *RAF*-mutated cells with resistance to TRAIL/DR5-mediated killing may survive in the circulation and eventually be able to metastasize to distal organs. Under such a scenario, elevated DR5 may favor the formation of metastasis-related signaling complexes such as the FADDosome as illustrated in Figure 1B and the enhancement of inflammation and cancer cell invasion and metastasis (Figure 3). This proposed connection also needs further investigation.

## 7. Summary and Perspectives

TRAIL/death receptor signaling has long been considered an apoptosis-inducing signaling cascade. However, it can also initiate other signaling pathways, unveiling non-apoptosis-related functions, including stimulation of inflammatory responses and cancer metastasis under certain circumstances. Hence, death receptor-mediated signaling complex proteins can serve as an important platform to regulate cancer apoptosis, inflammation, and metastasis depending on cell types or contexts. Under TRAIL-resistant or apoptosis-compromising conditions, activation of TRAIL/death receptor signaling may likely favor the positive regulation of cancer cell invasion and metastasis. Oncogenic Ras proteins such as K-Ras and H-Ras, in general, have shared redundant biological activities [100]. However, mDR or DR5 exert opposing effects on the regulation of cancer cell metastasis in murine tumorigenic models with different *RAS* mutations [40,56]. The underlying mechanisms accounting for this difference are largely unknown. Given the fact that there are two TRAIL death receptors named DR4 and DR5 in humans, whereas there is only one TRAIL death receptor (mDR) in mice [9,10] and the regulatory mechanisms of mDR expression are largely unknown in comparison with human DR5 expression, caution needs to be taken when extending the findings from murine models to the setting of human cancer.

TRAIL/death receptor-targeted therapeutics including recombinant proteins and agonistic antibodies have been developed for cancer therapy and are currently being tested in the clinic [12,13,101]. Unfortunately, although some positive trends have been observed with tolerable safety, no statistically significant anticancer activity has been achieved. Since the ultimate goal is to apply our knowledge to prevent metastatic dissemination during cancer therapy, including chemotherapy, targeted therapy, and immunotherapy, while simultaneously sensitizing cancer cells to die, we need a deep understanding of the biological functions of TRAIL/death receptor signaling in the regulation of cell death and metastasis. This will also inform appropriate strategies to design better therapeutics in the future for effective cancer therapy through targeting this critical signaling pathway for activating cell death and suppressing cancer cell invasion and metastasis. It is also important to distinguish TRAIL-sensitive from TRAIL-insensitive or resistant tumors so that we can apply tailored strategies for the treatment of specific cancer types with TRAIL/death receptor-targeting regimens while avoiding the stimulation of potential cancer cell invasion and metastasis.

Since resistance to TRAIL/death receptor-mediated apoptosis is a key factor associated with enhanced invasion and metastasis of cancer cells, it is critical to consider strategies aiming to target these cancer cells with resistance to TRAIL/death receptor-initiated apoptosis. Fortunately, many types of resistant cancer cells can be sensitized to TRAIL or death receptor activation-induced apoptosis by various means, particularly some small molecules that are able to increase death receptor expression and/or decrease the levels of c-FLIP and/or other anti-apoptotic proteins such as Mcl-1 [102,103]. Considering the heterogeneity of cancer cells, sensitive cancer cells may contain a subset of resistant cell populations that can escape TRAIL/death receptor-mediated killing. Therefore, combinatorial approaches offer a rational and actionable strategy to treat cancer to maximally eliminate cancer cells through the induction of apoptosis while minimizing the risk of increasing invasion and metastasis, which is often the reason for treatment failure.

## Data Availability

Not applicable.
